# The *SmERF1b-like* regulates tanshinone biosynthesis in *Salvia miltiorrhiza* hairy root

**DOI:** 10.1093/aobpla/plad086

**Published:** 2023-12-08

**Authors:** Dan Li, Yu Liu, Guoliang Chen, Yan Yan, Zhenqing Bai

**Affiliations:** College of Life Sciences, Yan’an University, Yan’an, Shaanxi 716000, China; College of Life Sciences, Yan’an University, Yan’an, Shaanxi 716000, China; College of Life Sciences, Yan’an University, Yan’an, Shaanxi 716000, China; College of Life Sciences, Yan’an University, Yan’an, Shaanxi 716000, China; College of Life Sciences, Yan’an University, Yan’an, Shaanxi 716000, China

**Keywords:** S. miltiorrhiza, SmERF1b-like, tanshinone

## Abstract

The ethylene response factor family genes are involved in the regulation of secondary metabolism in *Salvia miltiorrhiza*, but the mechanism underlying this regulation remains elusive. In the present study, based on the cDNA library of *S. miltiorrhiza*, an *AP2/ERF* gene was cloned and named *SmERF1b-like*. This gene exhibited a significant response to exogenous ethylene supply, such that ethylene remarkably upregulated *SmERF1b-like* expression levels in the leaves of *S. miltiorrhiza*. Subcellular localization showed that *SmERF1b-like* is located in the nucleus. Furthermore, *SmERF1b-like* showed a binding affinity with a GCC-box motif in the promoter region of genes associated with tanshinone biosynthesis in *S. miltiorrhiza*. Overexpression of *SmERF1b-like* in hairy roots of *S. miltiorrhiza* substantially upregulated *SmCPS1* and *SmKSL1* expression levels, resulting in increased biosynthesis of tanshinone I and cryptotanshinone contents. This finding provides valuable theoretical support for the utilization of a plant genetic engineering strategy to enhance *S. miltiorrhiza* resources.

## Teaser

The ethylene response factor (ERF) family genes play a key role in regulating secondary metabolism in *Salvia miltiorrhiz* a. Our study has identified *SmERF1b-like* as a nuclear-localized gene with the highest expression levels in leaves. We found that exogenous application of ethylene significantly triggers the response of *SmERF1b-like* . Furthermore, *SmERF1b-like* binds to the GCC-box motif in the promoter region of SmKSL1. Overexpression of *SmERF1b-like* substantially enhances the accumulation of tanshinone I (T-I) and cryptotanshinone (CT). In summary, *SmERF1b-like* promotes T-I and CT biosynthesis in *S. miltiorrhiza* by recognizing the GCC-box motif in the promoter region of SmKSL1. To further understand the molecular mechanism of *SmERF1b-like* in tanshinone biosynthesis, comprehensive multi-omic approaches and in-depth molecular-level studies are warranted. This study offers valuable theoretical insights for improving the genetic resources of *S. miltiorrhiza* and provides a reliable reference for resource selection and breeding strategies for this species.

## Background

AP2/ERF is one of the most extensive transcription factor (TF) families in plants, consisting of APETALA2 (AP2), ethylene response factor (ERF), ethylene response element binding protein (DREB), ABI3/VPI (RAV) and Soloist (Jofuku *et al.* 1994; Magnani *et al.* 2004; Mizoi *et al.* 2012; Gao *et al.* 2017; Zhang *et al.* 2018; Feng *et al.* 2020). Among these, ERF contains only one AP2/ERF domain, which comprises about 60 amino acid residues arranged into 3 anti-parallel β-sheets and an α-helix, with alanine at the 14th position and aspartic acid at the 19th position. The GCC-box (GCCGCC) is a cis-acting element that exhibits a specific affinity for ERF binding activity (Magnani *et al.* 2004; Mizoi *et al.* 2012; Gao *et al.* 2017; Zhang *et al.* 2018; Feng *et al.* 2020). The *ERF* genes play a pivotal role in plant growth and development, such as root development, stem elongation, leaf morphology, flower formation, fruit maturation, metabolic processes as well as responses to biotic and abiotic stresses (Xu *et al.* 2021). Additionally, ERFs have been identified as essential factors involved in modulating the biosynthesis of secondary metabolites in medicinal plants (Xiao *et al.* 2020). For instance, *AaERF1* and *AaERF2* upregulated the expression levels of *amorpha-4,11-diene synthase* (*ADS*) and *CYP71AV1*, thereby enhancing the accumulation of artemisinin in *Artemisia annua* (Barbacka and Baer-Dubowska 2011; Yu *et al.* 2012; Lu *et al.* 2013). *TcERF12* and *TcERF15* regulated taxol synthase gene expression, thus modulating the taxol biosynthesis pathway in *Taxus chinensis* (Zhang *et al.* 2015; Shao *et al.* 2021). *ORCA3* significantly improved the yield of indole alkaloids (TIA) in *Catharanthus roseus* (Zhu *et al.* 2015; Sun and Peebles 2016).

*Salvia miltiorrhiza* is a widely used medicinal plant in Traditional Chinese Medicine, while tanshinone as its main active compound is of great significance (Shan *et al.* 2021). The biosynthesis of tanshinones originates from two distinct pathways: the cytoplasmic mevalonate pathway and the plastid-localized methylene erythritol phosphate pathway (Wang and Wu 2010; Liu *et al.* 2015; Xiao *et al.* 2020; Zhan and Li 2020). *SmORA1*, an ERF gene, significantly promoted tanshinone accumulation in *S. miltiorrhiza* as this gene positively regulated the expression of key genes involved in tanshinone biosynthesis, including 3-hydroxy-3-methyl-glutaryl-CoA reductase 1 (*SmHMGR1*), 3-hydroxy-3-methyl-glutaryl-CoA reductase 2 (*SmHM- -GR2*), geranyl diphosphate synthase (*SmGPPS*), geranylgeranyl diphosphate synthase 1 (*SmGGPPS1*), *g*eranylgeranyl diphosphate synthase 3 (*SmGGPPS3*), copalyldiphosphate synthases 1 (*SmCPS1*), and kaurene synthase like 1 (*SmKSL1*) (Hua *et al.* 2016). The overexpression of *SmERF1L1* led to increased tanshinone accumulation and decreased salvianolic acid accumulation by upregulating 1-deoxy-d-xylulose-5-phosphate reductoisomerase (*SmDXR*), 1-deoxy-d-xylulose-5-phosphate synthase 2 (*SmDXS2*), *SmHMGS* and *SmKSL* expression levels in *S. miltiorrhiza* (Huang *et al.* 2019). Our team previously also reported that *SmERF6* and *SmERF8* bound with a GCC-box motif in promoter regions of *SmCPS1* and *SmKSL1*, thus promoting the accumulation of tanshinones in *S. miltiorrhiza* (Bai *et al.* 2018; Bai *et al.* 2020). *SmERF128* also showed the function of promoting the expression of *SmCPS1*, *SmKSL1* and *SmCYP76AH1*, leading to increased tanshinone accumulation in *S. miltiorrhiza* (Zhang *et al.* 2019). Conversely, *SmERF115* enhanced the expression of *S. miltiorrhiza* 4-couma royl-4ʹ-hydroxyphenyllactic acid (*SmRAS1*), which inhibited tanshinone synthesis and promoting salvianolic acid accumulation in *S. miltiorrhiza* (Sun *et al.* 2019).

In this study, the *SmERF1b-like* gene was identified as a potential TF through screening the *Salvia miltiorrhiza* cDNA library using the yeast one-hybrid (Y1H) technique (Bai *et al.* 2018). The open reading frame of *SmERF1b-like* was successfully cloned from the *S. miltiorrhiza* cDNA library. The bioinformatics analysis, gene characteristic evaluations and overexpressed *SmERF1b-like* hairy roots were employed to investigate the role of *SmERF1b-like* in tanshinone biosynthesis pathways in *S. miltiorrhiza*.

## Materials and Methods

### Experimental materials and treatments

The sterile seeds of *S. miltiorrhiza* were cultivated in MS medium for 45 d, and then the 45 d old seedlings were used in this experiment. The *S. miltiorrhiza* hairy roots induced by *Agrobacterium rhizogenes* (ATCC15834 strain) were from resources in our laboratory that were subcultured every 30 days. *Nicotiana benthamiana* plants were cultivated at a temperature of 25 °C with a 12 h photoperiod. Tissue-specific expression analysis was performed on the head, stem, leaf, flower and root samples of 2-year-old *S. miltiorrhiza*. These samples were collected, immediately frozen in liquid nitrogen, and stored at −80 °C. For ethephon treatment, 21-day-old *S. miltiorrhiza* hairy roots were shaken in liquid 6,7-V medium (25 °C, 120 rpm) and treated with a final concentration of 200 μg/L ethephon. Then, the treated samples were harvested separately at 0 h, 1 h, 2 h, 4 h, 8 h, 12 h, 24 h and 72 h, respectively. The untreated *S. miltiorrhiza* hairy roots grown under the same culture conditions were collected as controls. Each sample was subjected to three biological replicates, and after collection, the samples were frozen in liquid nitrogen, and then stored at −80 °C.

### Cloning of *SmERF1b-like* gene

The sequence information of *SmERF1b-like* gene (MH006601.1) was obtained from our previous research (Bai *et al.* 2018). The amplification primers were designed with primer 6.0. The primers are listed in  Supporting Information—Table S1. The cDNA of *S. miltiorrhiza* was used as the template. A 50 μL reaction system (25 μL 2xM5 HiPer plus Taq HiFi PCR mix, 2 μL upstream primer, 2 μL downstream primer, 2 μL cDNA template, 19 μL ddH_2_O) was used for amplification. The reaction conditions were: 95 °C, 5 min, 38 cycles (95 °C, 30 s, 55 °C, 30 s, 72 °C, 1 min), 72 °C, 10 min. The PCR product was separated by 1 % agarose gel electrophoresis, and the targeted gene fragment was purified and recovered using a gel recovery kit (TOLOBIO). The targeted gene fragment was ligated into pMD19-T simple cloning vector and then transformed into DH5α. Positive clones were screened on LB solid plates with 50 mg/L ampicillin at 37 °C for 12 h. Subsequently, positive clones were identified by PCR and then sequenced.

### Bioinformatics analysis of *SmERF1b-like* gene

The amino acid sequence of *SmERF1b-like* gene was obtained from the online program FGENESH (http://www.softberry.com/berry.phtml) and the gene structure was predicted. The characteristics of SmERF1b-like protein were analysed using ProtParam (http://web.expasy.org/protparam/). The protein secondary structure was predicted by SOPMA (http://npsa-prabi.ibcp.fr/cgi-bin/npsa_automat.pl?page=npsa_sopma.html). The protein tertiary structure was predicted by SWISS-MODEL (http://swissmodel.expasy.org/interactive). The signal peptide was predicted by SignalP 5.0 Serve (https://services.healthtech.dtu.dk/service.php?SignalP-5.0).

### Genomic DNA extraction, total RNA extraction and cDNA synthesis

DNA extraction from *S. miltiorrhiza* was conducted using the CTAB method (Bai *et al.* 2018). Total RNA of *S. miltiorrhiza* was extracted using an RNA extraction kit (Tiangen, China). The integrity of RNA was assessed, and then the RNA was reversed transcribed into cDNA using a PrimeScript^TM^ RT reagent kit with a gDNA Eraser kit (TaKaRa, China).

### qRT-PCR (quantitative real-time PCR) analysis

The qRT-PCR primers of *SmERF1b-like* were designed using Primer 6.0 ( Supporting Information—Table S1). *SmActin* was used as an internal reference gene. The reaction program comprised denaturing at 95 °C for 30 s, followed by annealing at 95 °C for 5 s and 60 °C for 30 s with 40 cycles using the SYBR Premix ExTaq (TaKaRa) kit and a BioRad CFX Manager 3.1 real-time PCR system.

### Subcellular localization analysis of *SmERF1b-like*

The full-length sequence of *SmERF1b-like* was recombined into PA70390 vector on the Nʹ terminal of the GFP-coding gene with the CaMV 35 S promoter downstream. Double restriction enzyme cutting sites XbaI and BamHI were used, and the primers are shown in  Supporting Information—Table S1. The PA70390-*SmERF1b-like* plasmid was infected into *Agrobacterium* strain EHA105, and then transiently expressed into tobacco leaves by injection. The injected tobacco seedlings were cultured overnight in the dark at 25 °C. Afterwards, protoplasts of the injected leaves were isolated using 1.5 % Cellulase R10 and 0.4 % Macerozyme. GFP fluorescence in the protoplasts was detected at 600 nm by laser confocal microscopy (Nikon A1R inverted confocal microscope) (Bai *et al.* 2018).

### EMSA verification

The induction and purification of MBP and SmERF1b-like protein were conducted as described in the previous study (Bai *et al.* 2018). SmERF1b-like protein expression was induced by 0.4 mM isopropyl-β-d-thiogalactopyranoside (IPTG). The designed and synthesized ERF protein recognition element DNA probe (CGCCGCC) was annealed, and after cooling at room temperature, the probe and purified SmERF1b-like protein were incubated at room temperature for 40 min using 6× loading buffer, and the colloid was placed in nucleic acid staining solution after completion and staining for 30 min. After rinsing 2–3 times, the gel was then placed under a chemiluminescence imaging system (ChemiDoc MP, BioRad) for photography and preservation, using the experimental method previously reported (Bai *et al.* 2018).

### Transgenic *S. miltiorrhiza* hairy roots analysis

The coding sequence of *SmERF1b-like* was digested and ligated with the PCAMBIA1304 vector. The overexpressed PCAMBIA1304-*SmERF1b-like* and PCAMBIA1304 plasmids were transformed into *Agrobacterium rhizogenes* ATCC15834. The positive clones were screened by YEB liquid plate with 50 mg/L kanamycin at 28 °C and 220 rpm in a shaking culture. PCAMBIA1304 and PCAMBIA1304-*SmERF1b-like* plasmids were used for plant transformation as previously described by our lab (Bai *et al.* 2018). DNA was extracted from transgenic hairy roots according to the instructions of the Plant Genomic DNA Extraction Kit (Tiangen, China). The transgene identification primers are shown in Supporting Information—Table S1.

### Tanshinone determination

The *S. miltiorrhiza* hairy roots were collected and dried at 45 °C for 3 days, and then ground into a fine powder. A total of 0.04 g dry powder was added to 8 mL of 70 % methanol for incubation at room temperature for 8 h. Subsequently, the mixture was centrifuged at 8000 rpm for 10 min. The supernatant was used for the detection of tanshinone compounds by high-performance liquid chromatography (HPLC) as previously described by Bai *et al.* (2018).

### Statistical analysis

Statistical analyses of HPLC data and qRT-PCR data were performed based on a one-way analysis of variance at *P* < 0.05 level using SPSS (version 13.0). All images were generated using EXCEL (version 2010) and TBtools software.

## Results

### Cloning and bioinformatics analysis of *SmERF1b-like* gene

**Figure 1.**
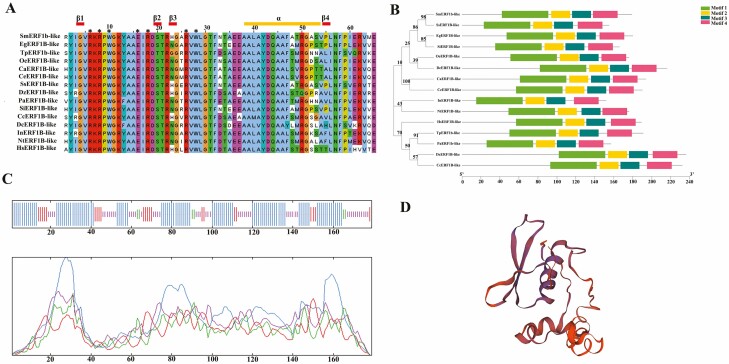
Gene structure and protein prediction of SmERF1b-like. (A) Conserved sequence of SmERF1b-like. (B) Phylogenetic tree and conserved domain of SmERF1b-like protein. SmERF1b-like: *S. miltiorrhiza*; EgERF1B-like: *Erythranthe guttata*; TpERF1B- like: *Trifolium pretense*; OeERF1B-like: *Olea europaea var. sylvestris*; CaERF1B-like: *Coffea arabica*; CeERF1B-like: *Coffea eugenioides*; SsERF1B-like: *Salvia splendens*; DzERF1B-like: *Durio zibethinus*; PaERF1B-like: *Prosopis alba*; SiERF1B-like: *Sesamum indicum*; CcERF1B-like: *Cynara cardunculus* var. *scolymus*; DsERF1B-like: *Daucus carota subsp. sativus*; InERF1B-like: *Ipomoea nil*; NtERF1B-like: *Nicotiana tabacum*; HsERF1B-like: *Hibiscus syriacus*. Different colours represent different conservative domains. (C) Secondary structure of SmERF1b-like. (D) Tertiary structure of SmERF1b-like.

The *SmERF1b-like* (MH006601.1) gene is composed of 540 base pairs, encoding a polypeptide chain consisting of 179 amino acids. The relative molecular mass of SmERF1b-like is 20.13 kDa (C867H1372N250O282S10), with an isoelectric point of 5.37. Its instability coefficient was calculated to be 65.15, while the average hydrophilic coefficient (GRAVY) value was −0.650, indicating that SmERF1b-like is a stable hydrophilic protein. Phylogenetic analysis revealed that SmERF1b-like exhibited the closest relationship with SsERF1B-like (XP042037389.1) (Fig. 1A and B). In terms of protein secondary structure, SmERF1b-like comprises 55.31 % α-helix, 3.35 % β-sheet, 29.05 % random coil and 12.29 % extended chain (Fig. 1C). The tertiary structure of SmERF1b-like was predicted using SWISS-MODEL (Fig. 1D), which confirmed the presence of an AP2 domain. PSORT prediction indicated that SmERF1b-like possesses a nuclear localization signal peptide. Therefore, it can be inferred that SmERF1b-like shares a similar function with SsERF1B-like. Notably, SmERF1b-like consists of four conserved sequences.

### Characterization analysis of *SmERF1b-like*

**Figure 2.**
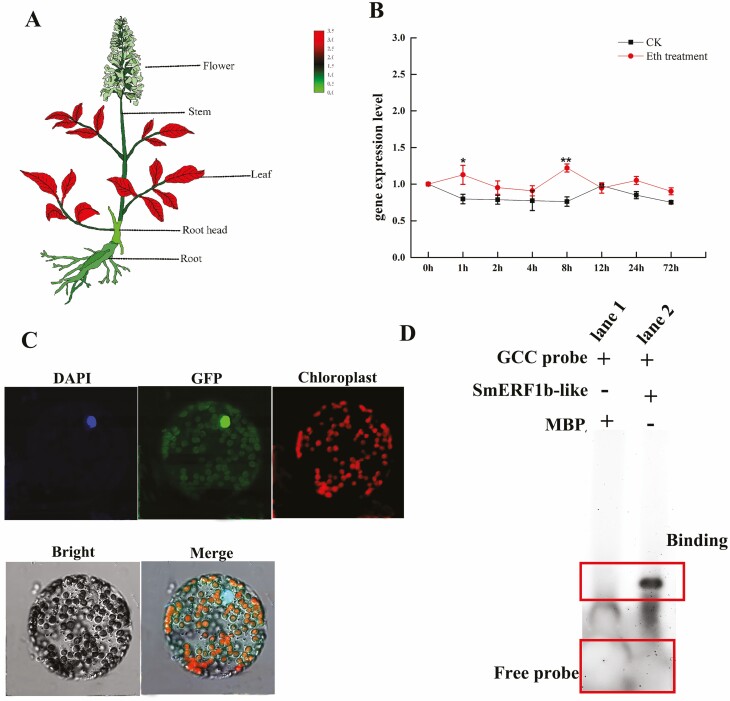
The characteristics of *SmERF1b-like*. (A) The expression levels of *SmERF1b-like* in root, leaf, flower, stem and root head (the joint between the stem and root). Data are shown as cartoon heatmap. (B) The gene expression levels of *SmERF1b-like* under ethylene treatment along with the time course. Eth indicates ethephon. Data are shown as the mean ± SD (*n* = 3), and the asterisk indicates the significance (*P* < 0.05) according to one-way ANOVA. (C) Subcellular localization of PA70390-SmERF1b-like. Merge: the overlap of DAPI, GFP, chloroplast and DT. DAPI, the nuclear marker; GFP, green fluorescent protein; chloroplast, chloroplast autofluorescence; bright, light field observations. (D) EMSA analysis: lane 1 has only the GCC probe (+) and no proteins (−). Lane 2 has only the GCC probe (+) and no proteins (−).

*SmERF1b-like* is a member of the ERF family, which may play a role in ethylene signalling. To analyse the expression of the *SmERF1b-like* in different tissues of *S. miltiorrhiza*, its expression levels in root head, stem, leaf, flower and root were detected. The findings revealed that *SmERF1b-like* expression was considerably higher in leaves than in other tissues (*P* < 0.05) (Fig. 2A). The gene expression level of *SmERF1b-like* was significantly increased at 1 h and 8 h exposure to ethylene treatment in *S. miltiorrhiza* hairy roots (Fig. 2B).

SmERF1b-like fused with GFP indicated a nuclear locational fluorescence signal. The GFP fluorescence of SmERF1b-like was also merged with DAPI, a nuclear marker, suggesting that SmERF1b-like localizes at the nucleus (Fig. 2C). Additionally, the GCC-box is a core cis-acting element involved in the recognition of ERF family genes. GCC-box sequence probes were used based on previous studies (Bai *et al.* 2018). The results of the EMSA showed clear protein and DNA binding bands when SmERF1b-like and the probe coexisted, demonstrating that SmERF1b-like protein can recognize the GCC-box (Fig. 2D).

### *SmERF1b-like* regulates the accumulation of tanshinones in *S. miltiorrhiza* hairy roots

**Figure 3.**
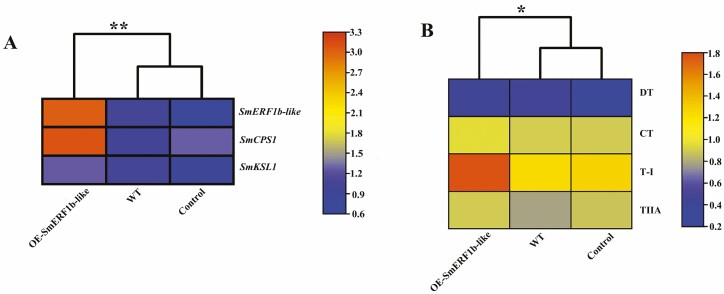
Overexpression of *SmERF1b-like* in *S. miltiorrhiza* hairy root. WT: ATCC-induced *S. miltiorrhiza* hairy root. Control: the pCAMBIA1304 empty vector counterparts. OESmERF1b-like represents the positive overexpressed *SmERF1b-like* hairy root lines. A, the gene expression levels of *SmERF1b-like*, *SmCPS1* and *SmKSL1* in WT, control and transgenic hairy root lines, respectively. (B) The tanshinone contents in WT, control and transgenic hairy root lines. DT-I, dihydrotanshinone; T-I, tanshinone I; T-IIA, tanshinone IIA; CT, cryptotanshinone.

To further elucidate the regulatory role of *SmERF1b-like* in tanshinone biosynthesis, we generated and analysed transgenic hairy roots with overexpression of *SmERF1b-like*. Positive transgenic lines were identified by PCR amplification using NPT, rolb, rolc and the targeted gene as positive markers. The line exhibiting the highest expression level of *SmERF1b-like* was selected for subsequent experiments. There was a substantial increase in both the expression levels of *SmERF1b-like* and the accumulation of tanshinone compounds, particularly tanshinone I (T-I) and cryptotanshinone (CT), in the *SmERF1b-like* overexpressed lines (Fig. 3A and B). The content of tanshinone IIA in the *SmERF1b-like* overexpressed plants was also significantly increased (Fig. 3B).

## Discussion

The AP2/ERF family genes are closely associated with the synthesis and regulation of active ingredients in medicinal plants (Liu *et al.* 2015). *SmERF1b-like* has 540 bp and encodes 179 amino acid residues. It contains α-helices, β-sheets and only one AP2 domain, exhibiting alanine (A) and aspartate (D) residues at positions 14 and 19 of the AP2 domain, respectively, which is consistent with the characteristics of the AP2/ERF family genes (Nakano *et al.* 2006; Licausi *et al.* 2010; Ji *et al.* 2016; Xu *et al.* 2021). *SiERF1b* is associated with drought resistance in *Sesamum indicum* (Dossa *et al.* 2016). Since SmERF1b-like sequence is very similar to *SiERF1b*, *SmERF1b-like* may also be involved in plant stress resistance (Nakano *et al.* 2006; Ji *et al.* 2016). Tanshinone, one of the primary active ingredients in *S. miltiorrhiza*, is synthesized in leaves and transported in roots for storage (Ji *et al.* 2016). The expression levels of *SmERF1b-like* were significantly higher in leaves than in other organs, suggesting that *SmERF1b-like* may be involved in tanshinone biosynthesis in *S. miltiorrhiza*. TFs mainly participate in metabolic regulation in the nucleus (Ren and Kong 2017). *SmERF1b-like* contains a nuclear localization signal peptide, and transient expression in tobacco leaves confirmed that SmERF1b-like is located in the nucleus. Moreover, exogenous application of ethylene significantly induced *SmERF1b-like* expression, indicating that it is a typical *ERF* family gene.

ERF TFs mainly bind to the GCC-box in the promoters regulating the downstream gene expression. *SmDXR*, *SmDXS2*, *SmHMGS*, *SmCPS1*, *SmKSL1* and *SmCYP76AH1* are key genes involved in tanshinone synthesis, while *SmERF1L1*, *SmERF6*, *SmERF8*, *SmERF128* and *SmERF2* regulate tanshinone synthesis by binding with the GCC-box of their promoters (Bai *et al.* 2018, 2020; Huang *et al.* 2019; Sun *et al.* 2019; Zhang *et al.* 2019). In this study, we found that *SmERF1b-like* regulates tanshinone synthesis by binding with the GCC-box in the promoter region of *SmKSL1*, which is a crucial gene involved in tanshinone biosynthesis. Moreover, overexpression of *SmERF1b-like* significantly increased the content of tanshinone I and cryptotanshinone, implying that *SmERF1b-like* binds with the GCC-box in the promoter region of key genes involved in tanshinone synthesis. As a result, upregulation of *SmERF1b-like* increases tanshinone accumulation.

## Conclusions

**Figure 4.**
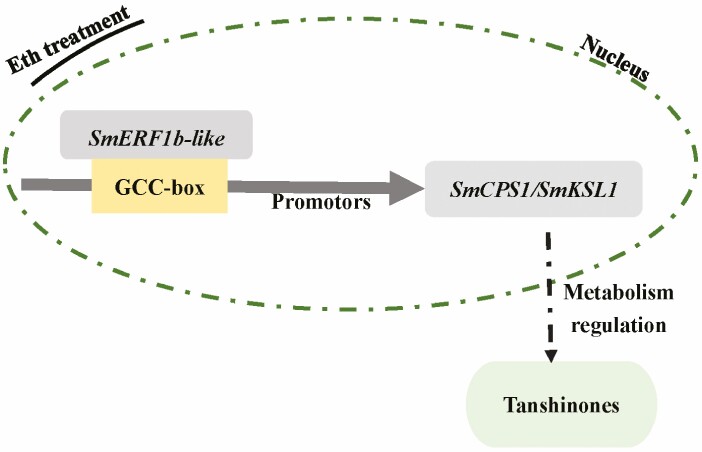
The role of SmERF1b-like in the regulation of tanshinone biosynthesis.

*SmERF1b-like* localizes in the nucleus and shows the highest expression levels in leaves. Exogenous application of ethylene remarkably induces the response of *SmERF1b-like*. Additionally, SmERF1b-like binds with the GCC-box motif in the promoter region of *SmKSL1*. The overexpression of *SmERF1b-like* substantially increases tanshinone I (T-I) and cryptotanshinone (CT) accumulation. Overall, *SmERF1b-like* is involved in promoting T-I and CT biosynthesis accumulation in *S. miltiorrhiza* by recognizing the GCC-box motif in the promoter region of *SmKSL1* (Fig. 4). To gain further insights into the molecular mechanism of *SmERF1b-like* in tanshinone biosynthesis, comprehensive multi-omic approaches and in-depth molecular-level studies are needed. This study provides valuable theoretical guidance for enhancing the genetic resources of *S. miltiorrhiza* and serves as a reliable reference for resource selection and breeding strategies for this species.

## Supporting Information

The following additional information is available in the online version of this article –

**Figure S1.** The protein purification and western blotting analysis of MBP and SmERF1b-like. M: marker.

**Table S1.** The primers for gene cloning, gene expression, vector construction and identification of *SmERF1b-like* used in this study.

plad086_suppl_Supplementary_Tables_S1

plad086_suppl_Supplementary_Figures_S1

## Data Availability

All relevant data are within the manuscript and its additional files.
