# Supplementary material for: The SmERF1b-like regulates tanshinone biosynthesis in Salvia miltiorrhiza hairy root
Source: AoB Plants. 2023 Dec 8;16(1):plad086. doi: 10.1093/aobpla/plad086 (PMC10799320; doi:10.1093/aobpla/plad086)
Supplement: plad086_suppl_Supplementary_Tables_S1 [file plad086_suppl_supplementary_tables_s1.docx]

| **Table S The primers used in the gene clone，gene expression,vector construction and identification of SmERF1b-like** | | | | |
| --- | --- | --- | --- | --- |
| **Primer names** | **5'-------------3'** | | **Restriction site** | **TM（℃）** |
| **SmERF1b-like-clone** | **Forwords sequence** | **TGAGATGGAGTCAGAGTCATGTTGG** |  | **55** |
|  | **Reserved sequence** | **GGTAGCTAGTATTGATGATTTGATGG** |  |  |
| **SmERF1b-like-QRT-PCR** | **Forwords sequence** | **CTTTAGGTGCGAGGAGGGCTCAT** |  | **60** |
|  | **Reserved sequence** | **CCTCTGCTTCTTCAACTGGCTTCT** |  |  |
| **SmERF1b-like-PMAL-C2x** | **Forwords sequence** | **GGGGAATTCATGGAGTCAGAGTCATG** | **EcoRI** | **59** |
|  | **Reserved sequence** | **GGGAAGCTTGCCTAGTATTGATGATTTG** | **HindIII** |  |
| **SmERF1b-like-GFP** | **Forwords sequence** | **GGGTCTAGACATGGAGTCAGAGTCATG** | **XbaI** | **59** |
|  | **Reserved sequence** | **GGGGGGATTCCTAGTATTGATGATTTG** | **BamHI** |  |
| **SmERF1b-like-1304** | **Forwords sequence** | **GGGACTAGTATGGAGTCAGAGTCATG** | **SpeI** | **59** |
|  | **Reserved sequence** | **GGGGGGACTAGTCTAGTATTGATGATTTG** | **SpeI** |  |
| **OE-identity-primer** | **Forwords sequence** | **GTGGATTGATGTGATATCTCCACTG** |  | **54** |
|  | **Reserved sequence** | **GGGGGGACTAGTCTAGTATTGATGATTTG** |  |  |
| **RNAi-identity-primer** | **Forwords sequence** | **GTGGATTGATGTGATATCTCCACTG** |  | **54** |
|  | **Reserved sequence** | **CCCTGATCATACCTCAGTCTCAGTAC** |  |  |
| **SmActin** | **Forwords sequence** | **GGTGCCCTGAGGTCCTGTT** |  | **60** |
|  | **Reserved sequence** | **AGGAACCACCGATCCAGACA** |  |  |
| **SmCPS1** | **Forwords sequence** | **CCACATCGCCTTCAGGG AAGAAAT** |  | **60** |
|  | **Reserved sequence** | **TTTATGCTCGATTTCGCTGCGATCT** |  |  |
| **SmKSL1** | **Forwords sequence** | **TGGGAACAGTGTGACCCTTCTGCT** |  | **60** |
|  | **Reserved sequence** | **GCTTGCATACAAATAACACCCAATCCT** |  |  |
| **rolB** | **Forwords sequence** | **GCTCTTGCAGTGCTAGATTT** |  | **55** |
|  | **Reserved sequence** | **GAAGGTGCAAGCTACCTCTC** |  |  |
| **rolC** | **Forwords sequence** | **CTCCTGACATCAAACTCGTC** |  | **55** |
|  | **Reserved sequence** | **TGCTTCGAGTTATGGGTACA** |  |  |
| **HptII** | **Forwords sequence** | **CGCTTCTGCGGGCGATTTGTG** |  | **55** |
|  | **Reserved sequence** | **GCTCTCGGAGGGCGAAGAATC** |  |  |
