# Supplementary figures and images for: The SmERF1b-like regulates tanshinone biosynthesis in Salvia miltiorrhiza hairy root
Source: AoB Plants. 2023 Dec 8;16(1):plad086. doi: 10.1093/aobpla/plad086 (PMC10799320; doi:10.1093/aobpla/plad086)

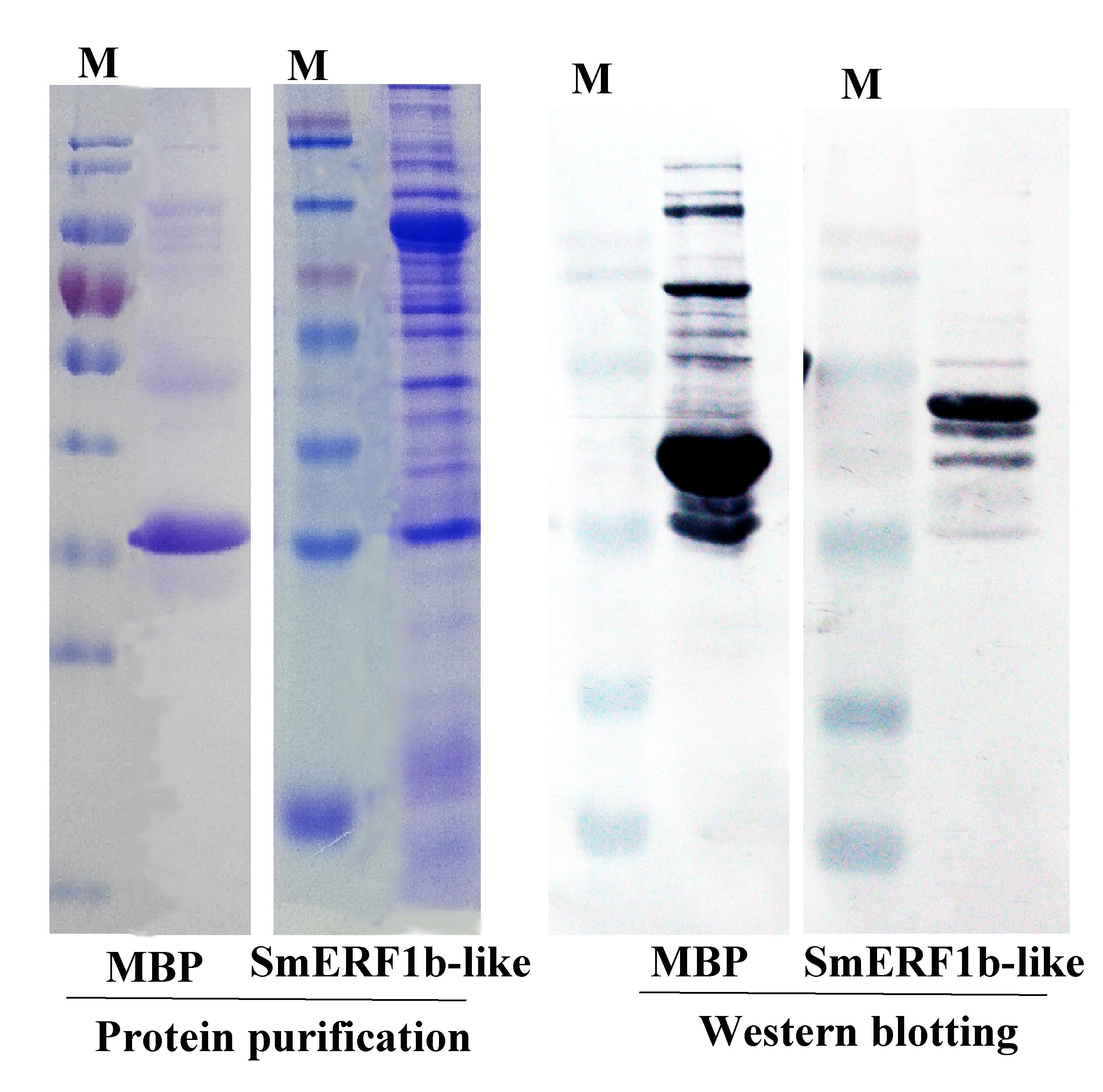

Supplement: plad086_suppl_Supplementary_Figures_S1 [file plad086_suppl_supplementary_figures_s1.jpeg]
